# Radiofrequency ablation with or without transarterial chemoembolization for hepatocellular carcinoma meeting Milan criteria: a focus on tumor progression and recurrence patterns

**DOI:** 10.3389/fonc.2024.1392495

**Published:** 2024-05-01

**Authors:** Yong Xie, Tianshi Lyu, Haitao Guan, Shoujin Cao, Li Song, Xiaoqiang Tong, Yinghua Zou, Jian Wang

**Affiliations:** Department of Interventional Radiology and Vascular Surgery, Peking University First Hospital, Beijing, China

**Keywords:** radiofrequency ablation, transarterial chemoembolization, hepatocellular carcinoma, Milan criteria, progression-free survival, local recurrence-free survival

## Abstract

**Background/objective:**

The aim of this study was to evaluate tumor progression and recurrence patterns of radiofrequency ablation (RFA) with or without transarterial chemoembolization (TACE) for treating hepatocellular carcinoma (HCC) that meets Milan criteria.

**Methods:**

This retrospective study included consecutive HCC patients meeting Milan criteria who underwent percutaneous RFA with or without TACE as initial treatment at a tertiary academic center between December 2017 and 2022. Technical success rate, local recurrence-free survival (LRFS), progression-free survival (PFS) and recurrence patterns were recorded.

**Results:**

A total of 135 HCC patients (109 male [80.7%]) with a mean age of 62 years and 147 target lesions were retrospectively enrolled. The technical success rate was 99.3%. The median LRFS was 60 months, and the cumulative 1-, 3-, and 5-year LRFS were 88.9%, 70.1%, and 30.0%, respectively. Additionally, the median PFS was 23 months, with cumulative 1-, 3-, and 5-year PFS of 74%, 30%, and 0%, respectively. Multivariate analysis confirmed that age > 60, alpha-fetoprotein (AFP) (> 10), and albumin were associated with PFS (2.34, p = 0.004; 1.96, p = 0.021; 0.94, p = 0.007, respectively). Six recurrence patterns were identified: local tumor progression (LTP) alone (n = 15, 25.0%), intrahepatic distant recurrence (IDR) alone (n = 34, 56.7%), extrahepatic recurrence (ER) alone (n = 2, 3.3%), IDR + ER (n = 2, 3.3%), LTP + IDR (n = 5, 8.8%), and LTP + IDR + ER (n = 2, 3.3%). IDR occurred most frequently as a sign of good local treatment.

**Conclusions:**

RFA in combination with TACE does not appear to provide an advantage over RFA alone in improving tumor progression in patients with HCC meeting the Milan criteria. However, further prospective studies are needed to confirm these findings and to determine the optimal treatment approach for this patient population.

## Introduction

Hepatocellular carcinoma (HCC) is one of the most prevalent malignancies worldwide and the leading cause of cancer-related deaths (1, 2), which varies greatly depending on the country and continent. Minimally invasive ablation has been well established as the first-line treatment option especially for cirrhotic patients with early-stage HCC, providing consistent local tumor control comparable to surgical resection, as recommended by relevant clinical guidelines (3–5). Additionally, patients with HCC meeting the Milan criteria are considered candidates for potentially curative therapies such as radiofrequency ablation (RFA) (6, 7).

Tumor progression can develop from residual cancer cells, intrahepatic distant recurrence (IDR), or extrahepatic recurrence (ER) after ablation, which adversely impacts patients’ survival outcomes (8, 9). Therefore, the achievement of a sufficient ablative safety margin has been widely recommended to reduce tumor progression or recurrence (9). The combination therapy of transarterial chemoembolization (TACE) and RFA has been recognized to allow the creation of larger ablation zones and reinforce anticancer effects when compared with RFA alone, leading to an improvement in tumor control and survival in patients with tumors measuring ≤ 5 cm (10). However, prior studies have reported that the differences in local tumor progression (LTP) and overall survival rates did not differ significantly in patients measuring ≤ 2 or 3 cm (11–13). Additionally, Huang et al. analyzed recurrence patterns in HCC patients less than 5 cm in diameter, but the inclusion of populations was not based on the Milan criteria (14). Therefore, the impact of these interventions on tumor progression and recurrence pattern in HCC patients meeting the Milan criteria needs further investigation due to several design limitations of previous studies (i.e., population selection focused on single lesions) (10–12).

This observational study aims to investigate the tumor progression rate and recurrence pattern in patients with HCC meeting the Milan criteria who underwent RFA alone or combined with TACE.

## Materials and methods

### Patients

This study received approval from the institutional review boards, which waived the requirement for written informed consent due to the retrospective nature of the study. Patients meeting the following criteria between December 2017 and 2022 were consecutively included: (i) diagnosis of HCC in accordance with relevant clinical guidelines (15); (ii) HCC meeting Milan criteria (single HCC lesion measuring ≤ 5 cm or up to three HCC lesions, each measuring ≤ 3 cm; without extrahepatic metastases or vascular invasion) (16); (iii) patients aged ≥ 18 years; (iv) patients who underwent percutaneous RFA with or without TACE as initial treatment; (v) Child–Pugh A or B liver function; (vi) Eastern Cooperative Oncology Group performance status (ECOG PS) 0. Exclusion criteria included: (i) patients with a history of previous treatments; (ii) patients with incomplete medical records; (iii) patients with a history of other malignancies; (iv) patients with inadequate follow-up data on tumor progression or outcomes.

### Data collection

The electronic medical records system was utilized to identify eligible patients. Important data, such as baseline demographic information, tumor characteristics, treatment specifics, and follow-up imaging findings, were retrieved from the medical records and inputted into a secure database.

### The procedures of RFA with or without TACE

Angiography of the abdominal vessels was performed using transfemoral access to identify tumor-feeding arteries, which were then catheterized superselectively using a coaxial microcatheter. Subsequently, TACE was performed by superselectively inserting microcatheters (Asahi Intecc Co., Ltd., Japan) into the tumor’s feeding arteries. Then, 10-30 mg of doxorubicin mixed with 5-10 mL of lipiodol (Guerbet, Villepinte, Seine-Saint-Denis, France) was injected slowly under continuous fluoroscopic guidance until blood flow in the tumor-feeding arteries ceased. The decision of whether to perform TACE before RF ablation depended not only on tumor factors (size, location, and poor conspicuity) but also on individual factors (such as patients’ wishes and economic affordability). In our center, we prefer TACE before ablation for tumors larger than 3 cm in diameter and for tumors with unfavorable ablation location (such as subphrenic HCC, adjacent to important organs such as vessels, gallbladder, or gastrointestinal tract). The time interval between TACE and RFA was usually within 4 weeks. RFA was performed under the guidance of ultrasound (US) or computed tomography (CT). Following the administration of local anesthesia (10-15 ml 2% lidocaine) and intravenous analgesia [remifentanil, 0.05ug/(kg.min)], a 2 or 3-cm expandable electrode (LeVeenTMSuperSlimTM, Needle Electrode System, Boston Scientific, US) was introduced into the target tumor under US guidance. The Boston Medical Device (RF 3000TM, Boston Scientific Way, Marlborough, MA 01752, US) was used according to the manufacturer’s standard recommendations for radiofrequency power settings and ablation times. Multiple overlap procedures using CT scans were performed during the RFA to ascertain the creation of a sufficient ablation zone. The puncture tract was ablated during electrode retraction to prevent bleeding or tract seeding. All procedures were performed by the same team consisting of six physicians with at least 10 years of experience in performing percutaneous RFA and TACE.

### Follow-up after RFA

A contrast-enhanced multiphase CT or magnetic resonance imaging (MRI) was conducted within 4 weeks following RFA to assess for any residual tumor. If residual tumor was present, immediate RFA was performed until the target lesion achieved complete ablation. If no evidence of residual tumor or HCC recurrence was found, follow-up was conducted every 2-3 months. Recurrent tumors in the ablated lesion or at other liver sites that emerged during follow-up were treated with the optimal treatment modality discussed by a multidisciplinary team, such as ablation, surgical resection, liver transplantation, TACE, systemic therapy, radiation therapy, or combination therapy.

### Definitions and data assessments

The primary outcome of interest was tumor progression, including LRFS and PFS, which was assessed based on radiological criteria such as tumor size, number of lesions, and intrahepatic or extrahepatic spread. LTP is defined as the appearance of enhancing HCC foci at the edge of the ablation zone (11, 17). PFS, including LTP, IDR, and ER, was measured by the interval from enrollment to either local or distant progression or death, whichever came first (18). Recurrence patterns include LTP alone, IDR alone, ER alone, or a combination of them. Technical effectiveness was defined as the complete ablation of the tumor on the contrast-enhanced multiphase CT or MR within 4 weeks following the initial RFA (17). Treatment effectiveness after RFA was determined according to the modified Response Evaluation Criteria in Solid Tumors (mRECIST) for HCC (19). However, overall survival was not considered as a primary outcome in this study, since OS will be evaluated in a later investigation. Patients were followed until December 2023.

### Statistical analysis

Descriptive statistics were utilized to summarize the baseline characteristics of the study population. Fisher’s exact test was employed to compare categorical variables, while the Mann-Whitney U test was used to compare continuous variables. The LRFS and PFS were calculated using the Kaplan–Meier method, and differences between groups were compared using the log-rank test. Cox proportional hazards regression analysis was conducted to identify factors associated with PFS. A p-value of less than 0.05 was considered statistically significant. In our study, all statistical analyses were conducted using R software, version 4.2.2 (https://www.r-project.org), in conjunction with MSTATA software (https://www.mstata.com).

## Results

### Patient characteristics

**Figure 1:**
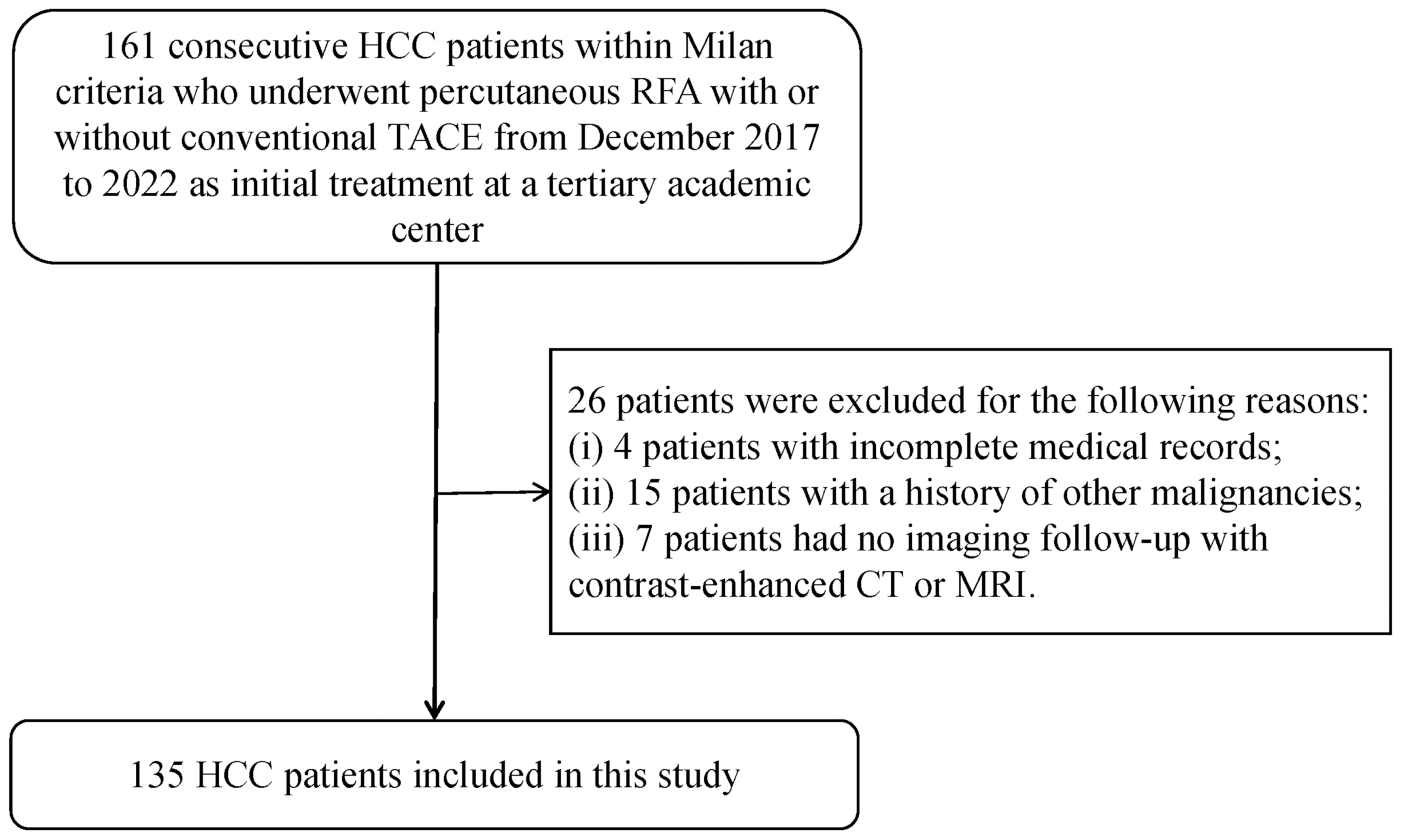
Patient selection of the study. HCC, hepatocellular carcinoma; RFA, radiofrequency ablation; TACE, transcatheter arterial chemoembolization; CT, computed tomography; MRI, magnetic resonance imaging.

One hundred and sixty-one patients who met the Milan criteria and received percutaneous RFA with or without TACE as the initial treatment were included in the study. Of these, 26 patients were excluded for the following reasons: 4 patients had incomplete medical records, 15 patients had a history of other malignancies, and 7 patients did not have imaging follow-up with contrast-enhanced CT or MRI. Ultimately, a total of 135 patients with 147 target lesions were included in this study (**Figure 1**). The 135 patients included 109 (80.7%) males and 26 (19.3%) females, with a mean age of 62 ± 10 years. Among them, 116 patients with 123 target HCC underwent TACE-RFA, while 19 patients with 24 target HCC underwent RFA monotherapy as the first-line treatment. The etiology of HCC (viral/nonviral), Child–Pugh score (A/B), presence of diabetes mellitus (yes/no), hypertension (yes/no), cirrhosis (yes/no), ascites (yes/no), number of tumors (single/multiple), tumor location (perivascular/subcapsular/subphrenic), maximum tumor size, Barcelona Clinic Liver Cancer (BCLC) stage (0/A), and some laboratory tests such as serum alpha-fetoprotein (AFP), serum albumin (ALB), and total bilirubin (TBIL) were also recorded. The characteristics of the patients and tumors are shown in **Tables 1**, **2**.

**Table 1:** Baseline characteristics of patients.

Characteristic	N = 135
Age (mean ± SD), years	62 ± 10
Age, n (%)	
≤ 60	61 (45.2)
> 60	74 (54.8)
Sex, n (%)	
female	26 (19.3)
male	109 (80.7)
Treatment type, n (%)	
TACE + RFA	116 (85.9)
RFA	19 (14.1)
Diabetes mellitus, n (%)	
no	100 (74.1)
yes	35 (25.9)
Hypertension, n (%)	
no	86 (63.7)
yes	49 (36.3)
Etiology, n (%)	
viral	97 (71.9)
nonviral	38 (28.1)
Cirrhosis, n (%)	
no	51 (37.8)
yes	84 (62.2)
Ascites, n (%)	
no	122 (90.4)
yes	13 (9.6)
No. of tumor, n (%)	
single	113 (83.7)
multiple	22 (16.3)
Maximum tumor size (mean ± SD), cm	2.23 ± 1.07
Maximum tumor diameter, n (%)	
≤ 3	106 (78.5)
> 3	29 (21.5)
Tumor location, n (%)	
single lobe	123 (91.1)
double lobes	12 (8.9)
BCLC stage, n (%)	
0	58 (43.0)
A	77 (57.0)
Child-Pugh score (IQR)	5.00 (5.00, 5.00)
Child-Pugh score, n (%)	
A	119 (88.1)
B	16 (11.9)
AFP (ng/ml), n (%)	
≤10	81 (60.0)
>10	54 (40.0)
Perivascular, n (%)	
no	90 (66.7)
yes	45 (33.3)
Subcapsular, n (%)	
no	66 (48.9)
yes	69 (51.1)
Subphrenic, n (%)	
no	89 (65.9)
yes	46 (34.1)
GGT (IQR), IU/L	37 (27, 71)
AST (IQR), IU/L	30 (22, 38)
ALT (IQR), IU/L	22 (16, 35)
ALB (mean ± SD), g/L	38.0 ± 5.6
TBIL (IQR), umol/L	17 (13, 23)
SCr (mean ± SD), umol/L	79 ± 23
PLT (IQR), ×10^9^/L	115 (76, 170)
PT (IQR), s	12.15 (11.60, 13.20)
INR (mean ± SD)	1.09 ± 0.13

SD, standard deviation; TACE, transarterial chemoembolization; RFA, radiofrequency ablation; BCLC, Barcelona Clinic Liver Cancer; IQR, interquartile range; AFP, alpha-feto protein; GGT, gamma-glutamyltransferase; AST, aspartate aminotransferase; ALT, alanine aminotransferase; ALB, albumin; TBIL, total bilirubin; SCr, creatinine; PLT, platelet; PT, prothrombin time; INR, international normalized ratio.

**Table 2:** Baseline characteristics of tumors.

Characteristic	N = 147
Age (mean ± SD), years	62 ± 11
Sex, n (%)	
female	29 (19.7)
male	118 (80.3)
Treatment type, n (%)	
TACE + RFA	123 (83.7)
RFA	24 (16.3)
Etiology, n (%)	
viral	105 (71.4)
nonviral	42 (28.6)
Couinaud segment location, n (%)	
S1	2 (1.4)
S2	16 (10.9)
S3	10 (6.8)
S4	18 (12.2)
S5	25 (17.0)
S6	21 (14.3)
S7	24 (16.3)
S8	31 (21.1)
Perivascular, n (%)	
no	102 (69.4)
yes	45 (30.6)
Subcapsular, n (%)	
no	75 (51.0)
yes	72 (49.0)
Subphrenic, n (%)	
no	99 (67.3)
yes	48 (32.7)
Tumor diameter (mean ± SD), cm	2.16 ± 1.06

SD, standard deviation.

### Technical success rate

According to the mRECIST criteria, we consider technical success to have been achieved if complete tumor necrosis is found on contrast-enhanced CT or MR within 4 weeks of the initial RFA. Upon initial treatment, the technical success rate was 99.3% (146/147), with only one target tumor requiring additional RFA due to residual unablated tumors identified by the contrast-enhanced CT within 4 weeks of the initial treatment, as a result of mistargeted ablation. The residual lesion was successfully treated with immediate additional RFA sessions.

### Local tumor response

**Figure 2:**
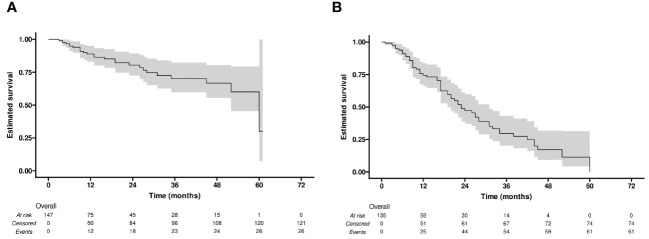
Kaplan-Meier curves of **(A)** local recurrence-free survival (LRFS), and **(B)** progression-free survival (PFS).

The median LRFS was 60 months (95% confidence interval [CI]: 52-NA). The LRFS rates for all 147 lesions were 88.9% at 1 year, 80.5% at 2 years, 70.1% at 3 years, 66.8% at 4 years, and 30.0% at 5 years (**Figure 2A**).

### Risk factors for PFS and systemic tumor response

Univariable and multivariable Cox regression analyses revealed that age >60 (hazard ratio [HR]: 2.34, 95% CI: 1.31-4.17, p = 0.004), AFP >10 (HR: 1.96, 95% CI: 1.11-3.46, p = 0.021) were associated with lower PFS, while ALB (HR: 0.94, 95% CI: 0.89-0.98, p = 0.007) was associated with longer PFS (**Table 3**).

**Table 3:** Results of univariate and multivariate Cox regression analyses of PFS.

Characteristic	Univariate analysis			Multivariate analysis		
	HR	95% CI	p-value	HR	95% CI	p-value
Sex						
female	—	—				
male	1.14	0.58, 2.25	0.707			
Age						
≤ 60	—	—		—	—	
> 60	2.16	1.27, 3.65	0.004*	2.34	1.31, 4.17	0.004*
Treatment type						
TACE + RFA	—	—		—	—	
RFA	0.99	0.39, 2.49	0.985	1.23	0.43, 3.46	0.699
Diabetes mellitus						
no	—	—				
yes	1.32	0.77, 2.27	0.308			
Hypertension						
no	—	—				
yes	1.64	0.96, 2.79	0.068			
Etiology						
nonviral	—	—				
viral	1.33	0.76, 2.32	0.318			
Cirrhosis						
no	—	—		—	—	
yes	1.24	0.74, 2.08	0.405	1.54	0.86, 2.76	0.143
Ascites						
no	—	—				
yes	1.45	0.57, 3.69	0.438			
No. of tumor						
single	—	—		—	—	
multiple	0.97	0.49, 1.91	0.919	0.63	0.28, 1.40	0.255
Maximum tumor size						
≤ 3	—	—		—	—	
> 3	2.07	1.19, 3.59	0.010*	1.09	0.52, 2.27	0.822
Tumor location						
single lobe	—	—				
double lobes	0.80	0.32, 2.02	0.640			
BCLC stage						
0	—	—		—	—	
A	2.12	1.20, 3.75	0.009*	1.81	0.85, 3.83	0.123
Perivascular						
no	—	—		—	—	
yes	0.86	0.50, 1.47	0.573	0.91	0.50, 1.67	0.758
Subcapsular						
yes	—	—		—	—	
no	0.64	0.38, 1.07	0.088	0.74	0.38, 1.43	0.366
Subphrenic						
no	—	—		—	—	
yes	1.52	0.90, 2.57	0.122	1.02	0.56, 1.85	0.946
Child-pugh score						
A	—	—				
B	1.51	0.65, 3.55	0.340			
AFP						
≤ 10	—	—		—	—	
> 10	1.67	1.00, 2.78	0.049*	1.96	1.11, 3.46	0.021*
GGT	1.00	0.99, 1.01	0.952			
AST	1.00	0.99, 1.01	0.712			
ALT	1.00	0.99, 1.01	0.790			
ALB	0.94	0.91, 0.98	0.006*	0.94	0.89, 0.98	0.007*
TBIL	1.00	0.97, 1.02	0.739			
SCr	1.01	1.00, 1.02	0.233			
PLT	1.00	1.00, 1.00	0.735			
PT	1.19	0.98, 1.43	0.072			
INR	7.44	0.86, 64.36	0.068			

PFS, progression-free survival; CI, confidence interval; HR, hazard ratio; TACE, transarterial chemoembolization; RFA, radiofrequency ablation; BCLC, Barcelona Clinic Liver Cancer; AFP, alpha-feto protein; GGT, gamma-glutamyltransferase; AST, aspartate aminotransferase; ALT, alanine aminotransferase; ALB, albumin; TBIL, total bilirubin; SCr, creatinine; PLT, platelet; PT, prothrombin time; INR, international normalized ratio. *p-value <0.05.

**Figure 3:**
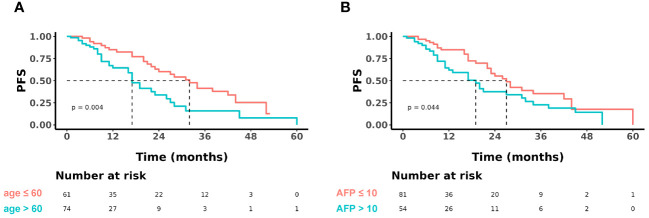
Kaplan-Meier curve of PFS comparing **(A)** the age ≤ 60 group versus the > 60 group, and **(B)** Alpha-feto protein (AFP) ≤ 10 group versus the > 10 group.

During the follow-up period (20.6 months in median, range: 1-65 months), 61 (45%) of the 135 patients developed tumor progression or recurrence, including one patient who died. The median PFS after RFA for all patients was 23 months (95% CI: 19-31). The cumulative 1-, 2-, 3-, and 4-year PFS rates were 74% (95% CI: 66-84), 47% (95% CI: 37-60), 30% (95% CI: 20-43), and 17% (95% CI: 9-32), respectively (**Figure 2B**). Furthermore, the cumulative PFS rates were 85.0% (95% CI: 75.3-96.0) at 1 year, 60.2% (95% CI: 46.5-77.9) at 2 years, 41.4% (95% CI: 27.9-61.6) at 3 years, 25.3% (95% CI: 13.4-47.6) at 4 years in the age ≤ 60 group, and 64.5% (95% CI: 52.4-79.5), 33.8% (95% CI: 21.2-54.0), 15.9% (95% CI: 6.4-39.4), and 7.9% (95% CI: 1.5-41.6) in the age > 60 group (p = 0.004) (**Figure 3A**). The median PFS in the age ≤ 60 group was 32 months (95% CI: 23-44), which was significantly longer than the 17 months (95% CI: 16-27) in the age > 60 group (**Figure 3A**). In addition, the cumulative 1-, 2-, 3-, and 4-year PFS rates after RFA with or without TACE were 84.9% (95% CI: 75.7-95.2), 55.2% (95% CI: 41.6-73.3), 35.3% (95% CI: 22.3-56.0), and 17.7% (95% CI: 7.0-44.5), respectively, in the AFP ≤ 10 group, and 62.0% (95% CI: 49.2-78.3), 37.6% (95% CI: 24.6-57.4), 22.8% (95% CI: 11.8-43.8), and 14.2% (95% CI: 5.6%-36.3), respectively, in the AFP > 10 group (p = 0.044) (**Figure 3B**). The median PFS in the AFP ≤ 10 group was 27 months (95% CI: 23-44), which was significantly longer than the 19 months (95% CI: 12-32) in the AFP > 10 group (**Figure 3B**).

### Recurrence pattern

**Figure 4:**
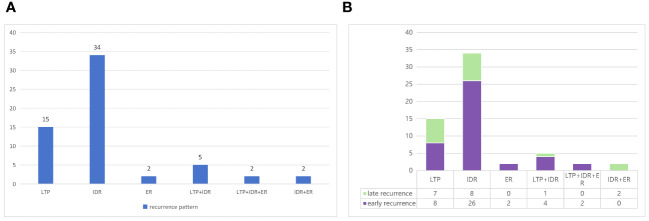
**(A)** Types of recurrence based on the location and **(B)** patterns of early or late recurrence in the study.

The recurrence pattern was divided into three types based on the location of the recurrence: LTP, IDR, and ER. Ultimately, six recurrence patterns were identified: LTP alone (n = 15, 25.0%), IDR alone (n = 34, 56.7%), ER alone (n = 2, 3.3%), IDR + ER (n = 2, 3.3%), LTP + IDR (n = 5, 8.8%), and LTP + IDR + ER (n = 2, 3.3%) (**Figure 4A**). IDR occurred most frequently as a sign of good locoregional treatment. In line with previous studies defining early or late recurrence (i.e., 2 years as the cut-off point) [20], the results of Fisher’s test showed no statistically significant difference in the recurrence pattern between the two groups (p = 0.054) (**Figure 4B**).

## Discussion

The present study demonstrates that RFA, with or without TACE, is effective for treating HCC that meets Milan criteria. However, tumor recurrence should not be ignored. Multivariable Cox analysis also shows that age, AFP, and ALB are significant risk or protective factors associated with PFS after RFA. Additionally, six recurrence patterns were identified.

**Figure 5:**
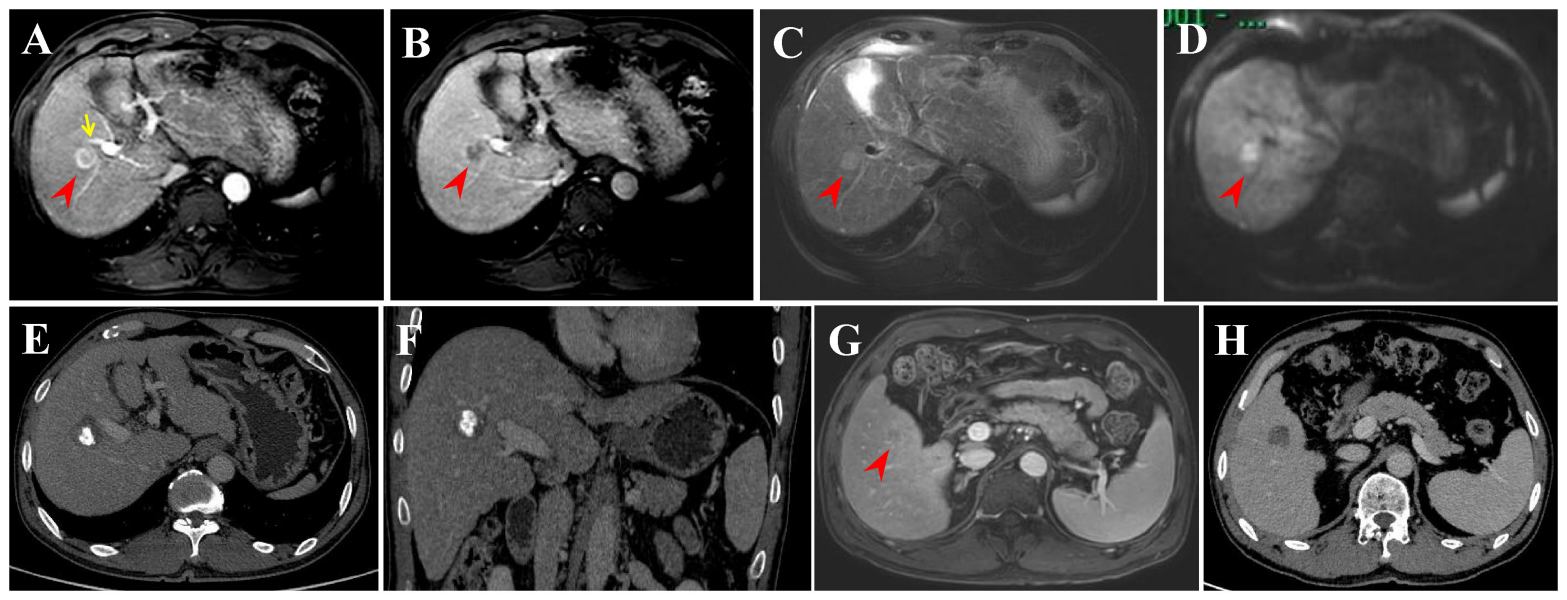
Intrahepatic distant recurrence (IDR) after transarterial chemoembolization (TACE) + radiofrequency ablation (RFA) for perivascular HCC in a 55-year-old man. **(A-D)** Dynamic contrast-enhanced axial magnetic resonance (DCE-MRI) scans show a small HCC (arrowheads) in periportal location (arrow) before treatment. **(E, F)** Contrast-enhanced computed tomography (CECT) scans obtained during portal venous phase 1 month after TACE + RFA showing the complete ablation zone adjacent to the portal vein. **(G)** DCE-MRI scans obtained during hepatic arterial phase (not shown) and portal venous phase 31 months after TACE + RFA showing the IDR (segment V). **(H)** CECT scan obtained during portal venous phase 1 month after RFA showing the complete ablation zone.

**Figure 6:**
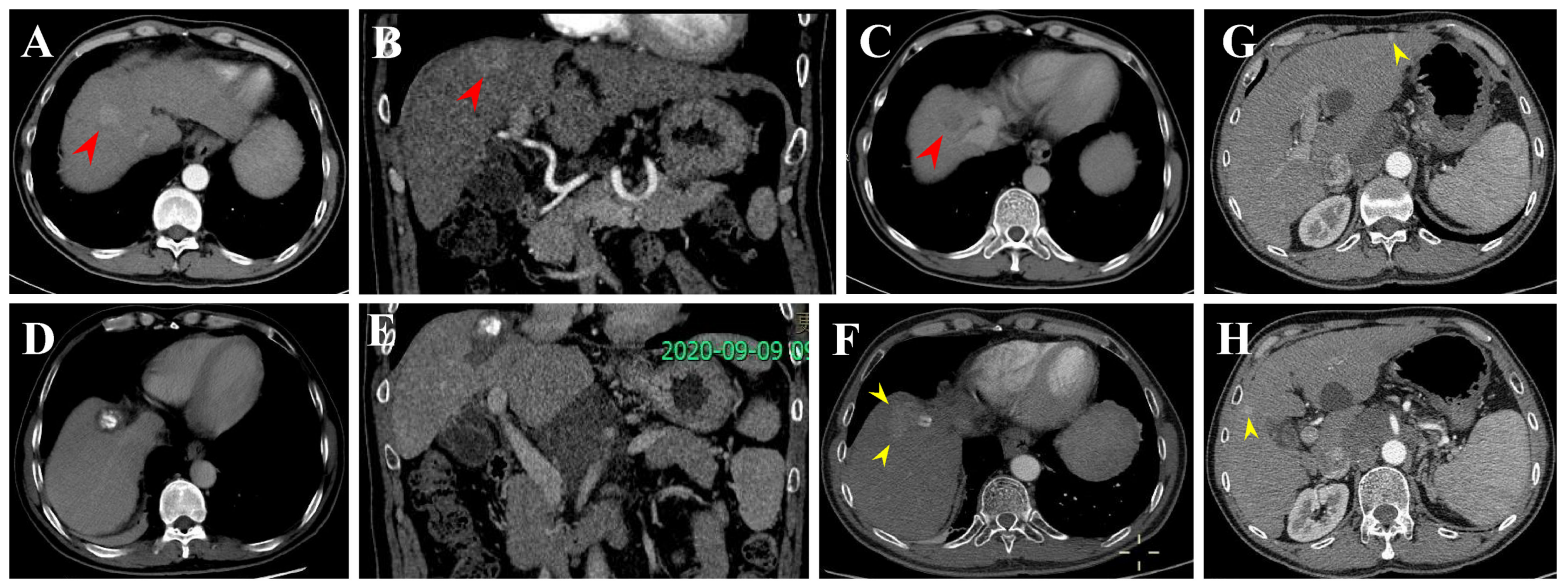
Local tumor progression (LTP) and IDR after TACE + RFA for subphrenic HCC in a 57-year-old man. **(A-C)** CECT scans show washin at hepatic arterial phase and washout at portal venous phase (arrowheads). **(D, E)** CECT scans obtained during portal venous phase 1 month after TACE + RFA showing the complete ablation zone adjacent to the diaphragm. **(F-H)** CECT scans obtained during portal venous phase 13 months after TACE + RFA showing the LTP and IDR (segment III and V). Then, the patient received TACE (not shown).

**Figure 7:**
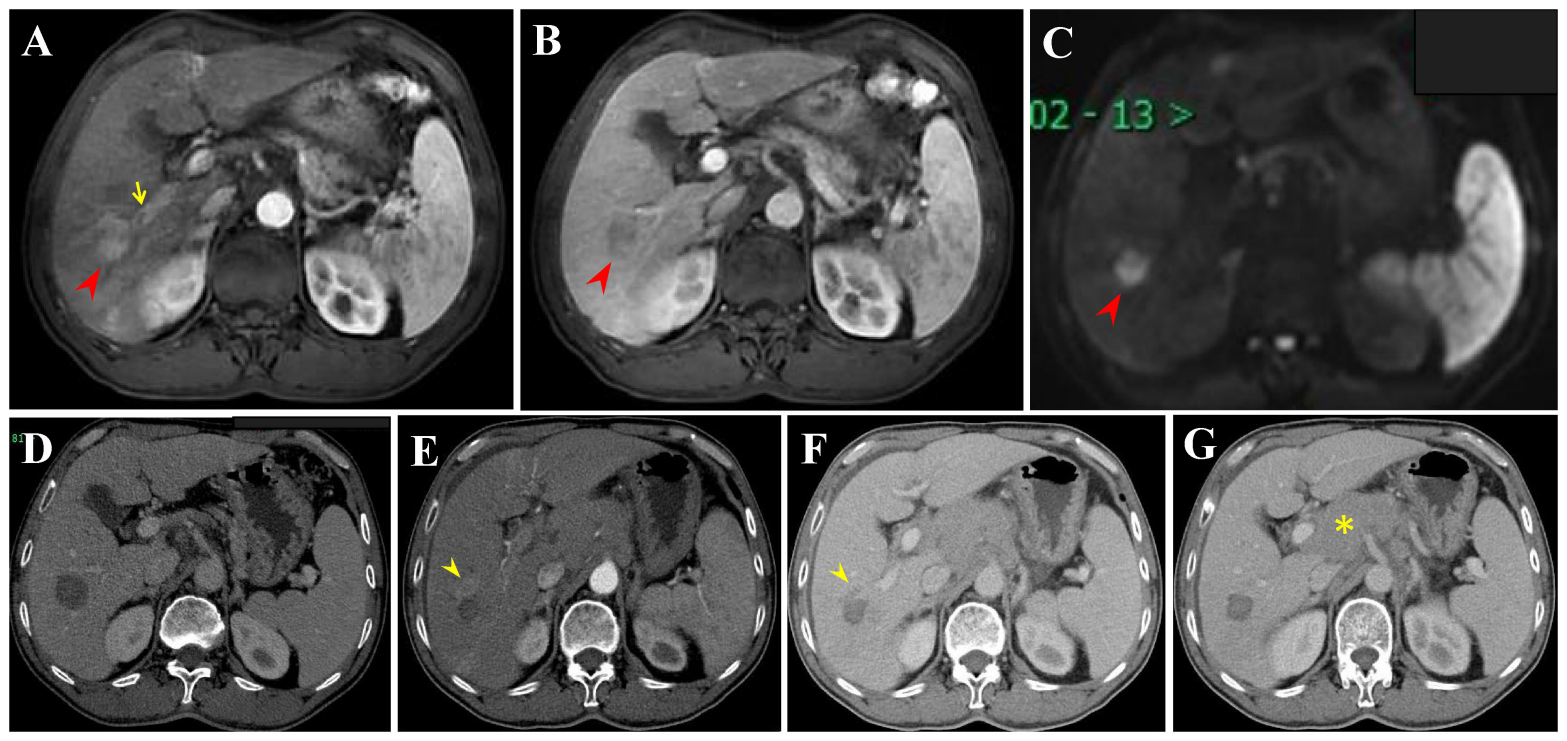
Extrahepatic recurrence (ER) and LTP after RFA alone for perivascular HCC in a 57-year-old woman. **(A-C)** DCE-MRI scans show washin at hepatic arterial phase and washout at portal venous phase (arrowheads). **(D)** CECT scan obtained during portal venous phase 1 month after RFA showing the complete ablation zone adjacent to the portal vein. **(E, F)** CECT scans obtained during hepatic arterial phase and portal venous phase 19 months after RFA showing the LTP, a small arterial enhancing nodule (arrowheads), with washout at portal venous phase. **(G)** CECT scan obtained during hepatic arterial phase (not shown) and portal venous phase 19 months after RFA showing the ER (asterisk). Then, the patient received locoregional theraphy (TACE + RFA) plus systemic treatment (not shown).

In this retrospective study, tumor progression or recurrence was observed in 61 HCC patients within the Milan criteria after RFA. Finally, six recurrence patterns were identified: LTP alone (25.0%), IDR alone (56.7%) (**Figure 5**), ER alone (3.3%), IDR + ER (3.3%), LTP + IDR (8.8%) (**Figure 6**), and LTP + IDR + ER (3.3%) (**Figure 7**). The most common type of recurrence was IDR, and the most common recurrence pattern was IDR alone, similar to previous studies (20, 21). Based on the specific time frame, the recurrence is categorized as either early or late, with a clear cut-off of 2 years. Patients who experience early recurrence have a poorer prognosis and may require more aggressive treatment options, such as liver transplantation or systemic therapy. In contrast, patients with late recurrence may benefit from surveillance strategies aimed at early detection and curative interventions (i.g., resection or ablation). These findings have important implications for treatment strategy decisions in patients with HCC. For patients at high risk of early recurrence, close monitoring and adjuvant therapy may be necessary to prevent disease progression and improve survival outcomes. In contrast, patients at risk of late recurrence may benefit from long-term surveillance and tailored treatment approaches based on tumor characteristics and underlying liver function. Understanding the patterns of HCC recurrence, whether early or late, is essential in guiding treatment decisions and improving patient outcomes. By identifying high-risk patients and implementing personalized treatment strategies, clinicians can optimize the management of HCC and improve long-term survival rates. Further research in this area is warranted to validate these findings and develop more effective therapeutic approaches for patients with recurrent HCC. However, those previous studies did not compare patterns of early or late recurrence. Our study further shows no statistically significant differences in patterns of recurrence between the two groups (i.e., early or late recurrence). To the best of our knowledge, this is the first retrospective study to compare patterns of early or late recurrence. Unfortunately, our study needs further analysis to explore its impact on survival. Considering the limited sample size of this study, further prospective studies are necessary to validate our results.

Several factors have been identified as potential predictors of LTP in HCC, including tumor size and location (e.g., perivascular, subcapsular, or subphrenic) (8, 22, 23). Understanding the factors associated with LTP is crucial for guiding treatment decisions and improving patient outcomes. Due to the limited number of people who develop LTP and the large number of confounding factors that need to be included, this study did not explore any independent factors affecting LTP. The frequency of LTP was 17.7% (26/147) [occurred at 3–60 months (mean: 18.8, standard error: 3.0, 95% CI: 12.6–25.1)], which is similar to previously reported rates of 9.6 to 27.3% (8, 9, 14, 21–24). Furthermore, the study found that the cumulative LRFS rates were 88.9% at 1 year, 80.5% at 2 years, 70.1% at 3 years, 66.8% at 4 years, and 30.0% at 5 years. Prior studies have reported cumulative rates of LTP at 1, 3, 5, 7, and 10 years as 6.6%, 14.9%, 20.4%, 22.6%, and 25.1%, respectively (8). The discrepancy between these studies may be due to differences in the HCC patients enrolled. In other words, the inclusion of larger HCC, tumor location (8, 22, 23), and number of tumors (25) might have adversely affected the LTP rate. Lee et al. confirmed that LTP is an independent risk factor for overall survival (8). Therefore, a safe ablation margin of 3 mm or more has been recognized as essential for preventing LTP and obtaining sufficient ablation (9). To create a sufficient ablation zone for lesions in high-risk recurrence sites, one strategy is to use TACE prior to RFA ablation to reduce the “heat-sink effect” (10, 26). TACE effectively reduces the inherent “heat-sink effect” of thermal ablation by blocking abundant blood flow in and around the tumor lesions. Additionally, the deposition of iodized oil or microspheres in the tumor can help heat conduction, better kill tumor cells, and cause tumor necrosis to achieve larger ablation zones (26–28).

**Figure 8:**
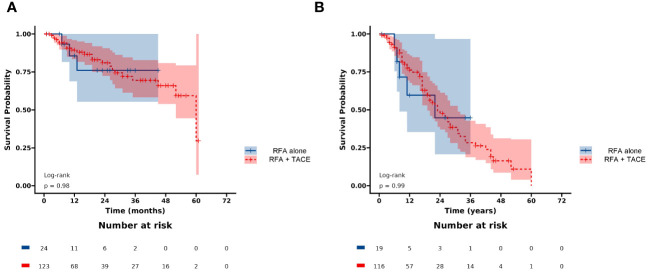
Kaplan-Meier curves of **(A)** LRFS, and **(B)** PFS between the RFA+TACE group and RFA alone group.

The BCLC staging system and AFP levels have been demonstrated to be effective in predicting the survival of HCC. Additionally, serum AFP plays an important role in the diagnosis of HCC, monitoring treatment response, and detecting disease recurrence after curative therapy. In the present study, multivariable Cox regression analyses showed that higher age (HR: 2.34, 95% CI: 1.31-4.17, p = 0.004), higher AFP (HR: 1.96, 95% CI: 1.11-3.46, p = 0.021), and lower ALB (HR: 0.94, 95% CI: 0.89-0.98, p = 0.007) were associated with lower PFS, which is consistent with previous studies (29–31). A multicenter study reported that elevated AFP levels were significantly associated with more microvascular infiltration (32). However, this can adversely affect PFS, which in turn affects the overall survival prognosis of patients (33). This result suggests that patients with high AFP levels may be prone to recurrence or progression, and close postoperative monitoring is urgently needed (31). The Child-Pugh grade is currently a commonly used tool to assess liver function and predict outcomes after HCC. ALB, as a component of the Child-Pugh grade, has been identified as a crucial prognostic factor for PFS in previous studies (25, 31, 34), which is similar to our present research. This suggests that good liver function may help reduce the risk of tumor progression, but further research is needed to confirm this. We also found there was no significant difference in LRFS in the combination of RFA and TACE group compared to RFA alone group (p = 0.98) (**Figure 8A**). In addition, there was no significant difference in PFS between the two groups (p = 0.99) (**Figure 8B**). Recent studies have shown that combining RFA with TACE may improve treatment efficacy and LTP compared to RFA alone in patients with HCC meeting Milan criteria. However, the effectiveness and safety of this combination therapy compared to RFA alone remain a topic of debate, with conflicting results reported in the literature (11–13). Therefore, the combination regimen does not appear to confer a greater advantage than RFA alone in improving tumor progression in this subset of patients. The takeaway is that combination therapy may not be necessary. However, it is expected that future randomized controlled studies will confirm this.

There are several limitations to the present study. Firstly, it is a retrospective study, which may introduce selection bias. Therefore, prospective, large-sample studies are necessary to verify our results. Secondly, due to the extended research period, advancements in RFA or TACE technology and concepts may have impacted the study results. Thirdly, the study population only included patients who underwent conventional TACE, excluding those who received doxorubicin-eluting bead-TACE. Lastly, the conclusions of this study may not be generalizable to other centers or ethnic groups due to differences in demographics and underlying causes of liver diseases.

## Conclusion

In conclusion, our retrospective study highlights the clinical significance of using RFA with or without TACE for HCC meeting Milan criteria. However, RFA in combination with TACE does not appear to provide an advantage over RFA alone in improving tumor progression. Moving forward, further investigations are warranted to validate the findings of our study and explore the long-term survival outcomes associated with RFA with or without TACE for HCC meeting Milan criteria. Future research should focus on comparing the safety of different treatment modalities, optimizing patient selection criteria, and assessing the cost-effectiveness of these approaches. Additionally, there is a need to investigate predictive factors for treatment response and develop personalized treatment algorithms for patients with HCC.

## Data availability statement

The original contributions presented in the study are included in the article/supplementary material. Further inquiries can be directed to the corresponding authors.

## Ethics statement

The studies involving humans were approved by Peking University First Hospital. The studies were conducted in accordance with the local legislation and institutional requirements. The ethics committee/institutional review board waived the requirement of written informed consent for participation from the participants or the participants’ legal guardians/next of kin because this was a retrospective study.

## Author contributions

YX: Data curation, Formal analysis, Resources, Software, Writing – original draft. TL: Data curation, Formal analysis, Resources, Software, Writing – original draft. HG: Investigation, Methodology, Writing – review & editing. SC: Investigation, Methodology, Writing – review & editing. LS: Investigation, Methodology, Writing – review & editing. XT: Investigation, Methodology, Writing – review & editing. YZ: Conceptualization, Supervision, Writing – review & editing. JW: Conceptualization, Supervision, Writing – review & editing.
